# Pelvic Floor Muscle Strength in the First Trimester of Primipara: A Cross-Sectional Study

**DOI:** 10.3390/ijerph19063568

**Published:** 2022-03-17

**Authors:** Lei Gao, Shiyan Wang, Di Zhang, Hongmei Zhu, Yuanyuan Jia, Haibo Wang, Suhong Li, Xiuhong Fu, Xiuli Sun, Jianliu Wang

**Affiliations:** 1 Department of Obstetrics and Gynecology, Peking University People’s Hospital, No. 11, Xi-Zhi-Men South Street, Xi Cheng District, Beijing 100044, China; 1911110389@bjmu.edu.cn (L.G.); 0062043910@bjmu.edu.cn (S.W.); 1911110388@bjmu.edu.cn (D.Z.); 2019112094@bsu.edu.cn (H.Z.); jiayuan@bjmu.edu.cn (Y.J.); wangjianliu@pkuph.edu.cn (J.W.); 2 The Key Laboratory of Female Pelvic Floor Disorders, Beijing 100044, China; 3 Department of Sports Medicine and Rehabilitation, Beijing Sports University, Beijing 100082, China; 4 Clinical Research Institute, Peking University, Beijing 100191, China; wanghb_pucri@bjmu.edu.cn; 5 Henan Key Laboratory of Fertility Protection and Aristogenesis, Luohe 462000, China; jp4217118173@qmul.ac.uk (S.L.); 1911210328@bjmu.edu.cn (X.F.); 6 Department of Obstetrics and Gynecology, Luohe Central Hospital of Henan Province, Luohe 462000, China

**Keywords:** pelvic floor muscle strength, primipara, pregnancy, sitting-toilet, squatting-toilet, the first trimester, levator hiatus

## Abstract

Background: Pelvic floor muscle (PFM) weakness is associated with stress urinary incontinence. Pregnancy is an important risk factor for PFM weakness. Studies evaluating PFM strength in the first trimester of pregnancy are still lacking. Our study aimed to describe pelvic floor function of the primipara in the first trimester of gestation and investigate the risk factors for PFM weakness. Methods: Primiparas aged 20~40 years with a singleton pregnancy less than 14 weeks of gestation were recruited, and data were collected via questionnaires on items that were suggested as associated with PFM weakness, followed by Modified Oxford Scale (MOS) on genital hiatus and perineal body and pelvic floor ultrasound evaluation for the thickness of the left and right levator ani muscles (LAM), right–left diameter of the levator hiatus (LH), and LH area. Participants were divided into three groups by MOS >3, =3, and <3 for data analysis. Results: A total of 380 participants completed the questionnaires and examinational analysis, of whom, 228, 98, and 54 were divided into Group 1, Group 2, and Group 3, respectively. The three groups were significantly different in the number of gestations and abortions, toilet types, and the right–left diameter of the LH (*p* < 0.05). Logistic regressive analysis showed that squatting toilet dominant (OR = 3.025; 95% CI: 1.623~5.638; *p* < 0.001) and a larger right–left diameter of the LH (OR = 1.065; 95% CI: 1.026~1.105; *p* = 0.001) were significantly associated with PFM weakness. Conclusions: Squatting toilet dominancy and longer right–left diameter of the LH are significantly associated with PFM weakness in primiparas in the first trimester. Sitting toilets should be recommended to women, especially pregnant women. Trial registration: The trial has been registered at Chinese Clinical Trial Registry (registration number: ChiCTR2000029618).

## 1. Introduction

Stress urinary incontinence (SUI) exerts a widespread negative impact on public health, making patients experience embarrassment and low self-esteem and become socially isolated [1,2]. The prevalence of SUI was reported to be 10~40% in women, the female population, 18~75% in late gestation, and approximately one-third in the postpartum period [3,4].

Weakened pelvic floor muscle (wk-PFM) plays an essential role in the development of SUI, anal incontinence, and pelvic organ prolapse (POP) [5]. Some experts [6,7] demonstrated that wk-PFM was associated with urinary incontinent (UI) in nulliparas. Wk-PFM was also a risk factor for SUI that could be modified during pregnancy [5]. PFM usually become relaxed together with the vaginal wall and the supportive tissues to ensure vaginal birth, which results from biomechanical and biochemical responses of the body to the relaxine and steroid hormones released during pregnancy [8,9], and accelerates the weakness of PFM. Comparative data showed that wk-PFM was related to UI in pregnant women [7].

PFM has been proved to be weakened during pregnancy [10]. Previous studies focused on PFM mainly in the second and the third trimesters of pregnancy as well as postpartum [11,12,13,14]. Santos et al. [15] measured the Modified Oxford Scale (MOS) of 76 primiparas in the second and the third trimesters via vaginal palpation, the median MOS was reported as 3 (1~4). As we all know, the uterus in the first trimester is smaller than the second and the third trimesters, thus, the pressure on PFM should be obviously different in those two pregnant stages. PFM strength in the second and the third trimesters is unable to represent that in the first trimester. Palmezoni et al. [10] analyzed 31 primiparas in the first trimester and 32 primiparas in the third trimester and concluded that MOS in the third trimester was lower than in the first trimester (2.5 vs. 2.1). Although the sample size was small, data from the previous studies indicated the risk of PFM weakness along with the progress of pregnancy. Weakened PFM was closely associated with the occurrence of SUI during pregnancy [7]. Furthermore, Palmezoni et al. [10] proposed that UI during pregnancy was a predictor of postpartum incontinence. Therefore, it is necessary to conduct large sample size studies to evaluate the PFM strength in the first trimester and explore its risk factors.

Women are worried about using perineometers in the vagina to measure PFM strength due to the pregnancy period. MOS is commonly used to evaluate PFM strength in gynecological examinations through manual vaginal palpation. Therefore, we adopt MOS in our study to evaluate PFM strength. To ensure study consistency, the particular assessors in each center were trained by the primary investigator (PI) in Peking University People’s Hospital.

Since pregnancy has been proved to be an independent risk factor of UI and the preventive indicators and measures of UI development should be studied on pregnant women [16] at young ages, we designed a study, Effect of the App-Based Video Guidance on Prenatal PFMT Combined with Global Postural Re-education for Stress Urinary Incontinence Prevention (PGT program) [17], to explore the preventive effect of antenatal pelvic floor muscle training (PFMT) on SUI at 6 weeks postpartum (the study). The study recruited primiparas ≤ 16 weeks of gestation to be randomized into training and control groups to perform PFMT under instruction and to have no training, respectively. Data collection via questionnaire interviews and clinic examinations were performed on both the groups and on the same schedule. As the baseline data collection on participants has been accomplished, we conducted an analysis of the data regarding to the PFM strength of primiparas in their first trimester to investigate the possible factors affecting the PFM strength, with anticipation to contribute convincing data for SUI preventive measures.

## 2. Materials and Methods

The study (PGT program) was collaboratively conducted by the investigators from 9 hospitals as the study sites in China Mainland from September 2020 to October 2021 following the published research protocol [17], on which the study sites were amended from 10 hospitals to be 9 after its publication. Primiparas in ≤16 weeks of gestation were recruited for participation. Data collected from the participants includes demographic characteristics, gynecological examination, PFM strength, and pelvic floor ultrasound. We conducted analysis on the data of the enrolled primiparas in the first trimester (<14 gestation weeks) of pregnancy to investigate the PFM strength, which, we believe, is closest to the pre-pregnant status.

The study was approved by the Ethics Committee of Peking University People’s Hospital (IRB number: 2019PHD107-02), the leading hospital, and was confirmed by the ethics committees of the other 8 hospitals as the study sites. Informed consent was obtained at each study site from all the participants in the study.

### 2.1. Participants

The participants were recruited from 9 hospitals in China mainland, including Peking University People’s Hospital, Peking University International Hospital, Peking University Shenzhen Hospital, Fangshan Maternal and Child Health Hospital of Beijing, Fengtai Maternal and Child Health Hospital of Beijing, Mentougou District Hospital of Beijing, Zhengzhou Central Hospital Affiliated to Zhengzhou University, Luohe Central Hospital of Henan Province, and The First Obstetrics Hospital of Shanghai. Primiparas were enrolled as the participants if they were 20~40 years of age with a singleton pregnancy at a gestation of less than 14 weeks. Participants were excluded if the pelvic examination for enrollment confirmed that they were (1) suffering serious complications including uncontrolled hypertension, diabetes mellitus, respiratory diseases, anorectal diseases, etc., (2) having pelvic floor dysfunction (PFD), SUI or fecal incontinence, POP, voiding or defecation dysfunction, and/or (3) had a history of gynecological surgery.

### 2.2. Measurement

All participants were requested to complete a questionnaire at the enrollment following the instruction of the investigators who were blind to the purpose of the questionnaire items. The questionnaires were designed to collect demographic characteristics including participants’ age, gestational weeks, educational status, pro-gestational body mass index (BMI) (within one month before pregnancy), occupation category, working posture, toilet type, PFMT habit, constipation history, smoking history, number of gestations, number of abortions, and family history of SUI and POP. We listed those items in the questionnaire to collect relevant data in referring publications, of which, each somewhat evidenced one or several of those items to be potentially associated with the PFM weakness. Definitions of some indicators are presented in Table 1. Following the questionnaire was collection of the data regarding the MOS, genital hiatus (gh), and perineal body (pb) by the urogynecologists according to the pelvic organ prolapse quantitation (POP-Q). Gh was the length between the external urethral orifice and hymen, while pb was the distance from the end of the gh to the middle of the anus during Valsalva maneuver. Valsalva maneuver is the performance of forced expiration against a closed glottis, such as the daily activity of straining during defecation.

The Modified Oxford Scale (MOS) is commonly used to evaluate PFM strength in gynecological examinations through manual vaginal palpation. In our study, each participant was examined for MOS by two urogynecologists (the examiner) and their scales were averaged as one in the record. The examiner first taught the participant how to contract and relax PFM without involvement of hip, thigh, or abdominal muscles. Then, the examiner inserted her/his middle and index fingers into participant’s vagina and instructed her to squeeze and lift the fingers up. If the participant could not contract the PFM involuntarily, she would be guided to perform the motion of interrupting urination to help them feel the PFM contraction. With the verbal encouragement of examiner, participant was requested to perform three contractions, each was separated by a period of rest. In requesting the participant to contract the PFM maximally, the examiner felt the PFM pressure against her/his fingers and scaled the MOS in six grades: grade 0 = no muscle contraction; grade 1 = flicker or pulsation; grade 2 = weak muscle contraction; grade 3 = moderate; grade 4 = good; and grade 5 = strong [18].

**Figure 1:**
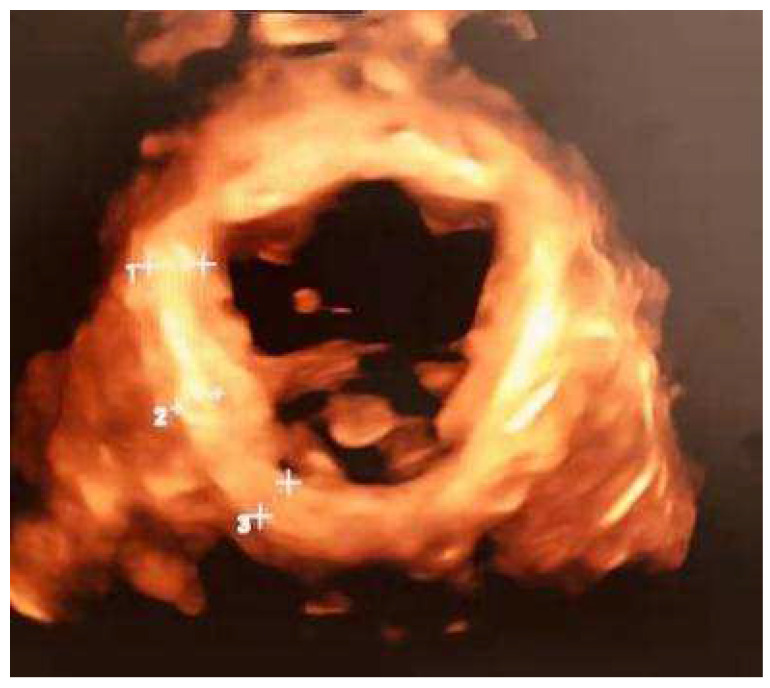
The measurement of the levator ani muscle (1 ++, 2 ++ and 3 ++ are the 3 points of levator ani muscle selected to measure on one side).

The ultrasound indicators were collected via transperineal ultrasound examination using 4D View v 10, a proprietary software of GE Kretz Medizintechnik (Shenzhen, China). Considering the participants were pregnant, images were collected at rest after having the participant lie in supine lithotomy position with hip flexing. The axial plane of minimal dimensions on the 4-dimensional ultrasound images was identified to calculate the levator hiatus (LH) area and measure the right–left diameter of LH. Finally, the thickness of the left and right levator ani muscles (LAM) was measured and recorded by averaging the values of the independent measurements at 3 points on each side. The space between the measuring points was determined to be 1 cm (Figure 1).

**Figure 2:**
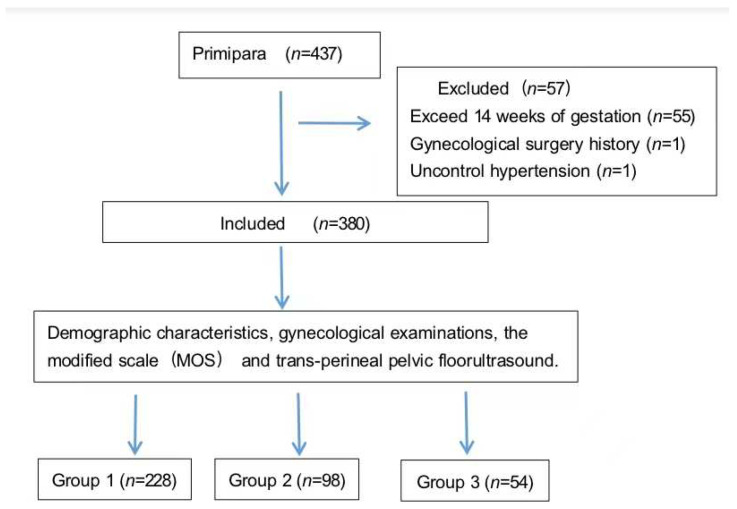
Study flow.

In data analysis, the participants were categorized into three groups as Group 1 if their MOS = 4 or =5, Group 2 if their MOS = 3, and Group 3 if their MOS = 0, =1, or =2 (Figure 2). Although we tried to investigate whether there was a difference between making three groups as MOS > 3, =3, and <3 or two groups as MOS > 3 and ≤3 before deciding how to group the participants and found it resulted the same as dividing either 3 or 2 groups, we still adopted the 3-grouping analysis with the consideration that MOS = 3 is the moderate PFM strength and should be referred to as a single indicator in participant grouping.

To ensure study consistency, urogynecologists who performed MOS and ultrasound physicians who conducted transperineal pelvic floor ultrasound in all 9 hospitals were trained by the PI from Peking University People’s Hospital. Data input devices were delivered to each hospital engaging in the study. Every hospital also prepared a paper file for each participant to record all collected data, which were stored in the relevant hospital.

### 2.3. Statistical Analysis

Statistical analysis was conducted using SPSS version 23.0 (SPSS, Inc., Chicago, IL, USA), and the assumed significance level was 5%. Categorical variables were described as numbers and percentages. Data were represented as the means ± standard deviations (SDs) when continuous variables followed a normal distribution. Otherwise, medians (P25, P75) were calculated when continuous variables did not follow a normal distribution. Univariate and multivariate regression analyses were performed to determine the association between PFM strength and age, gestational weeks, pre-gestational BMI, number of gestations, number of abortions, educational status, occupational category, toilet type, PFMT habit, constipation history, smoking history, family history of SUI, family history of POP, left LAM thickness, right LAM thickness, and the right–left diameter and area of the LH by ordinal logistic regression analysis. The results were presented as odds ratio (OR), adjusted OR, and 95% confidence interval (CI).

## 3. Results

A total of 380 eligible primiparas with mean age of 30 (28~32) years were enrolled, and their median gestational weeks was 12 (11~12). The demographic characteristics and gynecological examination indicators for all participants are described in Table 2. Among all the participants, 14 (3.7%) had ever performed satisfied PFMT. In total, 322 (84.7%) of the total had dominantly used sitting toilets for defecation, 54 (14.2%) used squatting toilets, and 4 (1.1%) reported not specified. The mean MOS of the primipara in the first trimester was 3.65 ± 1.058.

According to MOS, included in Groups 1, 2, and 3 are 228 (60%, 228/380), 98 (25.79% 98/380), and 54 (14.21% 54/380), respectively. The demographic characteristics and univariate analysis of the three groups are described in Table 3. The three groups were not significantly different in age, gestational weeks, pro-gestational BMI, educational status, working type, working posture, PFMT habit, constipation history, smoking history, family history of SUI, and family history of POP (*p* > 0.05), indicating that those elements have no impact on PFM. However, significant differences were observed among the three groups in number of gestations, number of abortions, and toilet type (*p* < 0.05).

Table 4 shows the correlation between PFM strength and transperineal pelvic floor ultrasound indicators. Statistical analysis showed that there were significant differences among the three groups in the right–left diameter of the LH (*p* < 0.05), but not in the left and right LAM thickness or the area of LH (*p* = 0.001), suggesting that those elements have no impacts on the MOS.

Logistic regression analysis on number of gestations, number of abortions, toilet type, and right–left diameter of the levator hiatus (Table 5) showed that squatting-toilet dominant (OR = 3.140; 95% CI: 1.810–5.448; *p* < 0.001) and a larger right–left diameter of the LH (OR = 1.055; 95% CI: 1.021–1.090; *p* = 0.002) were significantly associated with PFM weakness.

## 4. Discussion

PFM acts as a powerful pelvic stabilizer and provides support to pelvic organs. PFM injury will result in the pelvic organs being unable to be maintained in their normal positions [19]. Pregnancy and childbirth are known as risk factors for the development PFM weakness due to the increased pressure on PFM and the ruptures of PFM fibers, peripheral nerves, and connective tissues [20]. However, research on PFM strength based on a large number of cases of the first trimester primipara was lacking. Palmezoni et al. [10] recruited 31 primiparas in the first trimester and found that PFM strength measured by MOS was 2.5 ± 1.0. However, in our study, the mean PFM strength was 3.65 ± 1.058, which was higher than those reported from the previous study. To understand the difference between the two studies, we should also notice the facts that the inclusion criteria and the sample sizes of the two studies are also significantly different from each other. Our study also found 14.21% (54/380) primiparas with MOS ≤ 2, which indicated that there were some primiparas suffering from the weakened PFM needing to be given more attention.

Weakened PFM is closely related to SUI, fecal incontinence, POP, and sexual dysfunction [5,21]. Additionally, women’s quality of life and ability to engage in everyday activities may be negatively affected by PFM strength [20]. Blomquist et al. [5] analyzed 1143 participants after vaginal delivery and demonstrated that weakened PFM was associated with the cumulative incidence of POP, SUI, and overactive bladder. Martinez et al. [22] recruited 49 women to be interviewed for the Female Sexual Function Index questionnaire (FSFI) and graded for PFM strength, and concluded that women with stronger PFM were scored higher in the orgasm, sexual excitement, desire, and general questions on the questionnaire. Likewise, Sartori et al. [21] recruited 140 healthy females to evaluate the frequency of orgasm achievable sexual activities to objectively evaluate PFM strength, and found that better PFM was correlated with better sexual function, suggesting that strengthening PFM is hopefully resulting in improving women’s quality of life.

Many studies have proven that various factors, including age, parity, delivery method, and gynecological surgeries, affect PFM [2,23,24,25]. To decrease the influences of other confounding factors, we recruited participants in primiparas aged 20–40 years old without PFD and gynecological surgery in our study. Moreover, PFMT has been proved to increase PFM strength and endurance [26]. In reviewing 1701 articles, García-Sánchez et al. [27] suggested that 10–45 min of PFMT per session and 3–7 days per week might inspire the greatest changes in PFM. With understanding that such a frequent PFMT is hard for women to insist, we investigated PFMT in women’s daily life adopting once a week for 20 min with or without introduction of sports experts. It is also used to evaluate whether a feasible PFMT frequency is functional for PFM strength. Unfortunately, among the 14 participants who had PFMT at least 20 min per week in our study, no correlation was found between PFM strength and PFMT habit. A possible reason may be that PFMT for 20 min per week has no positive function on PFM strength. However, the group was too small for this to be a strong conclusion, which needs to be further proven by controlled study with a powerful number of cases.

The LAM is thought to be an important part of the pelvic floor and has been proven to play an essential role in preserving and supporting the function of pelvic organs [28]. The LH serves as an opening through the urethra, vagina, and rectum and passes the V-shaped LAM. The size of the LH is significantly associated with symptoms of POP and clinical signs [29]. A previous study showed a statistically significant correlation between the LH area and pelvic organ descent [30]. In our study, a statistically significant correlation was found: the longer the right–left LH diameter was, the higher the risk of suffering from weakened PFM was (OR = 1.055; 95% CI: 1.021–1.090; *p* = 0.002). Hoff et al. [31] proposed that supervised PFMT could increase muscle volume and close the LH. Literature and our clinical evidence all suggest that performing correct PFMT under instruction and supervision might exercise PFM to improve PFM strength and decrease the right–left diameter of the LH. Therefore, we will guide the participants in our study to perform correct PFMT under supervision in the coming study, with aim to investigate whether PFMT can improve PFM strength, decrease LH, and/or reduce SUI occurrence.

Our study also found that primiparas using sitting toilets dominantly had better PFM than those using squatting toilets. Rane et al. [32] found abdominal pressure was significantly higher in the squatting position than in the sitting position (*p* < 0.003), which is particularly prone to causing descent of pelvic floor organs and weakening of the pelvic stabilizer. There was a pilot study conducted by Rane et al. [32], in which they used 3D ultrasound to image the area of the LH and proposed that the levator hiatus is 9.5 cm^2^ larger on average in squatting position than in the supine position. These results indicate that the increased abdominal pressure together with the larger LH by using the squatting toilet may potentially have more negative impacts on the PFM. Over time squatting has a possibility of injuring PFM function and weakening PFM, which ultimately leads to a high risk of developing POP, SUI, dysuresia, and defecation dysfunction. To prevent chronic injury of the PFM, using a sitting toilet to replace squatting ones may greatly benefits women’s PFM according to our results.

Some experts [33,34,35] suggested that a squatting-based pelvic exercise regime accelerated the normal strengthening of the core muscle groups, including the muscles/ligaments of PFM. However, the only one study [33] verifying this was performed in children. We cannot extrapolate the results to adults. Furthermore, this does not contradict our result, since squatting-based pelvic exercise as a professionally designed posture focusing on stretching pelvic ligaments and contracting PFM to strengthen the PFM is totally different from defecation using a sitting toilet. When a woman squats on the toilet for a length of time, the increased abdominal pressure applied has negative impacts on the PFM.

The limitation of this study is that no baseline data before pregnancy was collected for comparison. Additionally, the PFM strength and right–left diameter of LH measured in the first trimester may not accurately represent the level before pregnancy due to the larger uterus and altered hormonal status. Considering the collagen changes and altered hormonal status (e.g., estrogen, progesterone, relaxin, etc.) during pregnancy and the individual anatomical differences, it is hard to verify whether the weakened PFM is physiological or pathological. Therefore, we will continue to carry out long-term follow-ups for all the participants until one year postpartum to investigate the risk factors for weakened PFM, expecting to provide convincing outcomes and to lay a foundation for primary prevention.

## 5. Conclusions

In conclusion, using a squatting toilet dominantly and a longer right–left diameter of the LH showed a significant association with weakened PFM in the trimester of the primipara. Using sitting toilets may potentially be important in prevention of the development of weakened PFM.

## Figures and Tables

**Table 1:** Measurement indicators impacting pelvic floor muscle and criteria for categorization.

**Occupational category**	Occupation in this study refers to a woman’s daily job. It was categorized according to the potential impacts of the job on the PFM as the follows: *Brainwork* refers to office work that had least impact on PFM. *Mild manual labor* refers to manual work in sitting position without heavy loads. *Medium manual labor* refers to manual work in standing position or moving with occasional heavy loads. *Heavy manual labor* refers to manual work with heavy loads that needs frequent or continuous body movement and high abdomen pressure to resist gravity.
**Working posture**	Refers to woman’s body posture when she is doing a job and is categorized according to the potential impacts of the job to the PFM as the follows: *Sitting* refers to a situation that a job is undertaken dominantly in sitting position. *Standing* refers to a situation that a job is undertaken dominantly in standing position. *Others* refers to a situation that a job is undertaken with posture shifting among sitting, standing, squatting, and bending, etc.
**Toilet type**	Refers to the type of toilet that a woman used for most of their defecating and is categorized according to the potential impacts on the PFM as squatting toilet dominant, sitting toilet dominant, or unspecified.
**PFMT habit**	Refers to whether a woman performed “satisfied PFMT”, which refers to PFMT at least once a week for 20 min in total in the past 3 months: *Yes* for performing satisfied PFMT in the past 3 months. *No* for not performing satisfied PFMT in the past 3 months.
**Constipation history**	Constipation refers to whether woman often felt defecation difficultly in the past 6 months and is categorized according to the potential impacts on the PFM as: *Yes* for often constipation in the past 6 months. *No* for recallable occasional constipation or no recallable constipation.
**Smoking history**	Refers to whether woman smokes or has ever smoked in her lifetime: *Yes* for current smoking or ever smoked. *No* for no smoking in her life.
**Number of gestations**	Refers to the number of gestation(s) a woman has had, including born, aborted, and in gestation.
**Number of abortions**	Refers to the number of abortion(s) a woman has had.

PFMT: pelvic floor muscle training.

**Table 2:** The demographic characteristics and gynecological examination indicators for all participants.

	All Participants
Age (years)	30 (28~32)
Gestational weeks	12 (11~12)
Pro-gestational BMI (kg/m^2^)	20.80 (19.30~22.68)
Toilet type	
Sitting-toilet dominant	322 (84.7%)
Squatting-toilet dominant	54 (14.2%)
Not specified	4 (1.1%)
PFMT habit	
Yes	14 (3.7%)
No	366 (96.3%)
POP-Q:	
Aa (cm)	−3.0 (−3.0~−3.0)
Ba (cm)	−3.0 (−3.0~−3.0)
Ap (cm)	−3.0 (−3.0~−3.0)
Bp (cm)	−3.0 (−3.0~−3.0)
C (cm)	−6.0 (−7.0~−6.0)
D (cm)	−7.5 (−8.0~−7.5)
Pb (cm)	3.0 (2.5~3.5)
Gh (cm)	3.0 (2.5~4.0)
TVL (cm)	8.0 (7.5~8.0)
MOS	
Grade 0	2 (0.5%)
Grade 1	9 (2.4%)
Grade 2	42 (11.3%)
Grade 3	98 (25.8%)
Grade 4	141 (37.1%)
Grade 5	87 (22.9%)
Mean grade	3.65 ± 1.058

BMI: body mass index; PFMT: pelvic floor muscle training; POP-Q: pelvic organ prolapse quantitation; Pb: perineal body; Gh: genital hiatus; TVL: total vaginal length; MOS: Modified Oxford Scale.

**Table 3:** Demographic characteristics and univariate analysis results for the three groups.

	Group 1 (*n* = 228)	Group 2 (*n* = 98)	Group 3 (*n* = 54)	OR (95% CI)	*p*
Age (years)	30 (28~32)	30 (28~32)	30 (28.5~32.5)	1.030 (0.971~1.091)	0.329
Gestational weeks	12 (11~12)	12 (11~12)	12 (11~13)	1.027 (0.909~1.160)	0.671
Pro-gestational BMI (kg/m^2^)					
<18.5	166 (72.8%)	65 (66.3%)	44 (81.5%)	1	
18.5–23.9	22 (9.6%)	20 (20.4%)	6 (11.1%)	1.461 (0.814~2.620)	0.204
24–27.9	27 (11.8%)	10 (10.2%)	4 (7.4%)	0.747 (0.379~1.470)	0.398
≥28	13 (5.7%)	3 (3.1%)	0	0.319 (0.087~1.167)	0.084
Number of gestations	1 (1~2)	1 (1~1)	1 (1~1)	0.631 (0.428~0.930)	0.020
Number of abortions	0 (0~1)	0 (0~0)	0 (0~0)	0.698 (0.494~0.986)	0.041
Educational status					
Graduate degree	45 (55.6%)	21 (25.9%)	15 (18.5%)	1	
Undergraduate	165 (60.2%)	73 (26.6%)	36 (13.1%)	0.787 (0.486~1.274)	0.330
High school	14 (70.0%)	3 (15.0%)	3 (15.0%)	0.560 (0.202~1.550)	0.264
Secondary education	4 (80.0%)	1 (20.0%)	0	0.282 (0.029~2.721)	0.274
Occupational category					
Brainwork	156 (61.4%)	62 (24.4%)	36 (14.2%)	1	
Mild manual labor	67 (57.8%)	33 (28.4%)	16 (13.8%)	1.123 (0.728~1.733)	0.599
Medium manual labor	5 (50.0%)	3 (30%)	2 (20.0%)	1.578 (0.477~5.225)	0.455
Heavy manual labor	0	0	0	-	-
Working posture					
Sitting-posture dominant	190 (59.6%)	85 (26.6%)	44 (13.8%)	1	
Standing-posture dominant	18 (54.5%)	8 (24.2%)	7 (21.2%)	1.331 (0.669~2.645)	0.415
Others	20 (71.4%)	5 (17.9%)	3 (10.7%)	0.609 (0.264~1.402)	0.244
Toilet type					
Sitting-toilet dominant	207 (64.3%)	78 (24.2%)	37 (11.5%)	1	
Squatting-toilet dominant	20 (37.0%)	18 (33.3%)	16 (29.6%)	3.139 (1.820~5.412)	<0.001
Not specified	1 (25%)	2 (50%)	1 (25%)	3.722 (0.605~22.897)	0.156
Family history of SUI					
Yes	6 (54.5%)	2 (18.2%)	3 (27.3%)	1	
No	149 (62.1%)	56 (23.3%)	35 (14.6%)	0.623 (0.198~1.958)	0.418
Unclear	73 (32%)	40 (31.0%)	16 (12.4%)	0.723 (0.225~2.321)	0.585
Family history of POP					
Yes	0	0	0	-	-
No	156 (62.4%)	62 (24.8%)	32 (12.8%)	1	
Unclear	72 (55.4%)	36 (27.7%)	22 (16.9%)	1.347 (0.889~2.042)	0.160
Constipation history					
Yes	21 (65.6%)	8 (25%)	3 (9.4%)	1	
No	207 (59.5%)	90 (25.9%)	51 (14.7%)	1.345 (0.636~2.845)	0.438
Smoking history					
Yes	4 (66.7%)	2 (33.3%)	0	1	
No	224 (59.9%)	96 (25.7%)	54 (14.4%)	1.559 (0.276~8.808)	0.615
PFMT habit					
Yes	8 (57.1%)	4 (28.6%)	2 (14.3%)	1	
No	220 (60.1%)	94 (25.7%)	52 (14.2%)	0.906 (0.319~2.574)	0.906

SUI: stress urinary incontinence; POP: pelvic organ prolapse.

**Table 4:** Univariate analysis of transperineal pelvic floor ultrasound indicators for the two groups.

	Group 1 (*n* = 228)	Group 2 (*n* = 98)	Group 3 (*n* = 54)	OR (95% CI)	*p*
Left LAM thickness (mm)	6.50 (5.70–7.60)	6.35 (5.40–6.80)	6.50 (6.20–7.78)	0.994 (0.864–1.143)	0.930
Right LAM thickness (mm)	6.60 (5.80–7.500)	6.50 (5.50–6.93)	6.60 (6.60–7.63)	1.055 (0.917–1.215)	0.453
Right–left diameter of LH (cm)	3.80 (3.40–4.00)	3.80 (3.60–4.10)	3.80 (3.70–3.94)	1.056 (1.023–1.091)	0.001
Levator hiatus area (cm^2^)	13.55 (11.90–15.66)	13.55 (11.61–14.83)	13.55 (12.35–14.07)	0.949 (0.889–1.013)	0.113

LAM: levator ani muscle; LH: levator hiatus.

**Table 5:** Ordinal logistic regression analysis of pelvic floor muscle strength.

	*p*	OR	95% CI
Number of gestations	0.465	0.729	0.312–1.703
Number of abortions	0.788	0.902	0.425–1.916
Toilet type			
Sitting-toilet dominant		1	
Squatting-toilet dominant	<0.001	3.140	1.810–5.448
Not specified	0.114	4.575	0.694–30.144
Right–left diameter of LH	0.002	1.055	1.021–1.090

## Data Availability

The data presented in this study are available on request from the corresponding author. The data are not publicly available due to the privacy of participants.
